# Development and validation of PRE-FRA (PREdiction of FRAilty risk in community older adults) frailty prediction model

**DOI:** 10.3389/fpubh.2025.1593668

**Published:** 2025-06-27

**Authors:** Taiping Lin, Xiaotao Huang, Xiang Wang, Miao Dai, Jirong Yue

**Affiliations:** ^1^Department of Geriatrics and National Clinical Research Center for Geriatrics, West China Hospital of Sichuan University, Chengdu, Sichuan, China; ^2^Department of Gastroenterology, Jiangyou Hospital, Mianyang, Sichuan, China; ^3^Jiujiang CityKey Laboratory of Cell Therapy, Department of Cardiology, Jiujiang NO.1 People’s Hospital, Jiujiang, Jiangxi, China; ^4^Jiujiang CityKey Laboratory of Cell Therapy, Department of Geriatrics, Jiujiang NO.1 People’s Hospital, Jiujiang, Jiangxi, China

**Keywords:** frailty, prediction model, older adults, community-dwelling, Lasso regression, nomogram, validation

## Abstract

**Background:**

As the global population ages, identifying older adults at risk of frailty becomes increasingly important for targeted interventions. This study aimed to develop and validate a 1-year frailty onset prediction model for initially non-frailty or pre-frailty, community-dwelling older adults.

**Methods:**

We enrolled 1,079 community-dwelling older adults aged >60 years without baseline frailty (i.e., non-frailty or pre-frailty) for the development cohort. Lasso regression was used to screen potential predictors. Subsequently, logistic regression analysis was conducted to create a nomogram, which was internally validated using 500 bootstrap resamples. Additionally, temporal validation was performed to ensure the model’s generalizability. This validation involved an external cohort of 481 older adults, all aged over 60 years and without frailty at baseline. Discrimination was assessed using the area under the receiver operating characteristic curve (AUROC), and calibration was evaluated with calibration plots.

**Results:**

In the development cohort, we enrolled 1,079 older adults with a median age of 68.00 years (interquartile range: 64.00–72.00), including 673 females. Over a 1-year follow-up, 73 cases of frailty were identified. Key predictors identified by the model included age, history of falls within the past month, coughing while drinking water, pre-frailtyty status, cognitive impairment, 5-time chair stand test, and calf circumference. The developed model exhibited favorable discriminative ability in the development cohort (AUROC = 0.81, 95% confidence interval 0.76–0.87). Internal validation through bootstrapping yielded consistent results (AUROC = 0.80), while temporal validation confirmed its robustness (AUROC = 0.73). Calibration plots demonstrated favorable agreement in both the development and temporal validation cohorts. To enhance usability, an online web-based calculator was developed (accessible at: https://frailtyriskprediction.shinyapps.io/dynnomapp/). The model showed high sensitivity (0.92) for frailty exclusion at a 2.5% threshold and specificity (0.89) for frailty identification at a 15% threshold.

**Conclusion:**

This 1-year frailty onset prediction model for initially non-frailty or pre-frailty older adults integrates accessible variables and demonstrates robust validation. It aids clinical decision-making by identifying high-risk individuals for early intervention.

## Background

The global demographic landscape is undergoing a profound transformation with a significant increase in the aging population. According to the World Health Organization, the number of individuals aged 60 years and older is expected to reach 2.1 billion by 2050, accounting for approximately 22% of the world’s population (1). While this demographic shift signifies advancements in healthcare, it also poses challenges, particularly in identifying and managing frailty-a multidimensional syndrome characterized by decreased physiological reserves and increased vulnerability to adverse health outcomes (2, 3), linked to disability (4), falls (5), hospitalization (6), and mortality (7, 8). Frailty is a dynamic state that may be mitigated through early intervention (9, 10), underscoring the need for accurate prediction tools. However, existing frailty prediction models exhibit critical limitations that hinder their clinical utility.

Current models predominantly rely on cross-sectional designs, which fail to capture the dynamic progression of frailty over time. For instance, models developed for specific populations-such as individuals with diabetes (11), heart failure (12), or hospitalized older adults (13, 14)-lack generalizability and often suffer from methodological limitations, including insufficient validation (11, 13–15) or narrow predictor selection (16). Although some prospective studies have attempted to address these gaps, their models frequently omit key domains (e.g., cognitive or physical performance measures) (17, 18), Although some prospective studies have attempted to address these gaps, their models frequently omit key domains (e.g., cognitive or physical performance measures) (AUROC≤0.72) (17, 19), focus narrowly on specific subpopulations (e.g., patients with malnutrition) (20), or lack rigorous validation (17, 18, 20, 21). Additionally, some prospective studies fail to distinguish between non-frailty and pre-frailty baseline status-a critical distinction for targeted prevention (17, 21). While online frailty calculators offer a convenient means of estimating predictive variable probabilities, research in this area remains limited.

Our study addresses these gaps by developing and validating a 1-year frailty onset prediction model specifically for initially non-frailty or pre-frailty community-dwelling older adults, integrating three innovative aspects: (1) longitudinal assessment of both traditional (e.g., age, pre-frailty status) and understudied predictors (e.g., calf circumference, coughing while drinking); (2) rigorous internal and temporal validation using multicenter data from western China, a region underrepresented in frailty research; and (3) the creation of a clinically actionable web-based calculator to facilitate real-time risk stratification. By focusing on the critical pre-frailty transition period and incorporating objective functional measures, our model advances beyond prior work to provide a practical tool for early intervention. This approach aligns with recent calls in gerontology for dynamic, multidomain frailty assessments while addressing the unmet need for validated, population-specific prediction tools in resource-limited settings.

## Methods

### Populations used to develop the prediction model

The 1-year PRE-FRA frailty risk prediction model (PREdiction of FRAilty Risk in community older adults) was developed utilizing data from the West-China Health and Aging Trend (WCHAT) study (22). The WCHAT is a comprehensive, observational, multicenter cohort study that began in 2018 across over 30 communities spanning four provinces in western China, with annual follow-up examinations planned. It has enrolled a diverse cohort of adults aged 50 years or older, employing a multi-stage cluster sampling strategy to ensure representation across various ethnic groups and considering the unique topographical features of the regions involved. Ethical approval for the WCHAT cohort study was granted by the Sichuan University Review Board at West China Hospital. Detailed information on the cohort profile of the WCHAT study may be found elsewhere (22, 23).

For the development of our prediction model, we utilized data collected from the WCHAT study between 2021 and 2022. Data from the period of 2018 to 2019 in the WCHAT study were reserved for temporal validation. The WCHAT study enrolled adults aged ≥50 years, but this analysis focused on community-dwelling participants aged ≥60 years without baseline frailty (who voluntarily completed all required assessments and measurements) to target older adults at highest risk of frailty progression. Participants were excluded if they (1) were lost to follow-up, (2) lacked frailty assessment data, or (3) were frail at baseline. Only non-frailty and pre-frailty participants were retained for model development and validation. The participant flow diagram (Supplementary Figure 1) details the screening process and exclusion reasons. The final analytic sample comprised 1,079 participants in the model development cohort and 481 in the temporal validation cohort.

### Assessment of frailty

The primary outcome was incident frailty (transition from non-frailty or pre-frailty at baseline to frail at 12-month follow-up). We utilized the Fried frailty phenotype (FFP) to define frailty (24), encompassing five distinct criteria detailed in Supplementary Table 1. Participants who did not meet any of the FFP criteria were classified as “robust” or “non-frailty.” Those meeting one or two criteria were labeled “pre-frailty,” while individuals meeting three to five criteria were identified as “frail” (24). Due to the relatively low incidence of frailty, the study population was divided into third groups: robust, frailty and non-frailty. The frailty diagnosis components were evaluated at baseline and throughout the follow-up period.

### Candidate predictors

All questionnaire investigations and anthropometric measurements were conducted and supervised by trained medical students in the form of face-to-face interviews, following the established protocol. Due to the limited number of outcome events and the necessity for a simple prediction model, baseline predictors were carefully selected. Potential predictor variables for frailty were identified from recent high-quality systematic reviews (25–28) and cohort studies (29–33). A panel of experienced, multicenter geriatricians were brought together to discuss prespecified candidate predictors. Ultimately, 33 potential frailty risk factors were incorporated into the development of our frailty prediction model, encompassing demographic details, anthropometric indices, and assessments of chronic illnesses. To reflect the real-world context of clinical epidemiology, each predictor was independently ascertained, separate from the primary outcome measure. These factors were meticulously evaluated at both baseline and during the 12-month follow-up period. For further detail, Supplementary Table 2 offers clear definitions and categorizations of the candidate predictors.

### Handling of missing data

In both the development and temporal validation cohorts, the percentage of missing data on predictors was relatively insignificant, as presented in Supplementary Table 3. Missing data (≤6.2% for all variables; see Supplementary Table 3) were imputed using multiple imputation by chained equations (MICE) with predictive mean matching for continuous variables and logistic regression for binary variables. Five imputed datasets were generated, and results were pooled using Rubin’s rules (34). This approach ensures that the model accounts for potential biases introduced by missing data, thereby enhancing the robustness of our findings. To assess the presence of potential bias among participants excluded due to loss of follow-up, we conducted a comparative analysis of baseline characteristics, comparing individuals who did not follow up with those who actively participated in the follow-up process (Supplementary Table 4).

### Statistical analysis

Baseline characteristics were summarized as counts and percentages for categorical variables and medians with interquartile ranges (IQR) for continuous variables, as appropriate for their distribution. Lasso regression (least absolute shrinkage and selection operator) was used to screen 33 prespecified candidate predictors. The tuning parameter (*λ*) was selected via 10-fold cross-validation to minimize the mean squared error (35, 36). We carefully evaluated the Lasso regression results to ensure that the selected predictors were both statistically significant and clinically relevant. Multivariable logistic regression was applied to the Lasso-selected predictors. Model assumptions (linearity, independence, absence of multicollinearity) were verified using variance inflation factors (VIF < 10) and tolerance values (>0.1). The final model adhered to the 10 events-per-variable (EPV) rule to prevent overfitting (37, 38). We have integrated the independent risk factors for incident frailty into our frailty risk prediction model. To facilitate interpretation, we have created a nomogram that visually represents the model, assigning a score to each variable (39). The cumulative ‘total points’ from the nomogram reflect the sum of the individual scores for the variables included, correlating to a 1-year probability of frailty onset, as indicated at the bottom of the nomogram. Subsequently, we developed a freely accessible online calculator to automate the prediction of the likelihood of frailty development in older adults over 12 months. The statistical analyses were performed utilizing R software (version 4.2.2; R Foundation for Statistical Computing).

### Model performance

We evaluated the predictive accuracy of our model using the area under the receiver operating characteristic curve (AUROC), determining the model’s ability to differentiate between participants who developed frailty during the follow-up and those who remained non-frailty (40). Predictive performance was classified as low (AUROC < 0.7), moderate (0.7 ≤ AUROC < 0.9), or high (AUROC ≥ 0.9) (41). Calibration plots were also created to assess the consistency between observed and predicted risks of frailty onset. Calibration plots and slopes were generated per TRIPOD guidelines, with slopes near 1 indicating optimal agreement (40). Furthermore, to assess the clinical applicability of our predictive model for decision-making, we conducted a decision curve analysis. This analysis evaluates the clinical utility of the model by considering both the benefits and potential harms associated with its use in clinical practice (42). Decision curve analysis (DCA) quantified the net benefit of the PRE-FRA model across threshold probabilities (4–40%), comparing it to ‘treat-all’ and ‘treat-none’ strategies. Net benefit reflects the trade-off between true positives (frailty prevention) and false positives (unnecessary interventions), with higher values indicating greater clinical utility.

### Model validation

We thoroughly evaluated the robustness of our prediction model through internal and temporal validation methods. For internal validation, we utilized the bootstrap resampling technique, creating 500 bootstrap samples. In each sample, the model was refitted to calculate the optimism-adjusted performance metrics, including the corrected area under the receiver operating characteristic curve (AUROC) (43). The corrected AUROC, a direct outcome of this process, served as a crucial indicator of potential overfitting in the original model (43). In the temporal validation, we assessed the model’s stability using a different cohort of participants. This aligns with TRIPOD guidelines for prediction model validation (44). The external validation cohort included 481 older adults, all aged over 60 years and without frailty at baseline. We adjusted the regression coefficients with participant-specific data to estimate the probability of frailty development and measured the model’s discrimination and calibration in this cohort (45).

### Sensitivity analysis

We conducted sensitivity analysis exclusively on complete cases and evaluated the potential influence of the multiple imputation approach on our research outcomes. The AUROC values obtained by only considering participants with complete data on candidate predictors exhibited predictive performance comparable to that of the overall analyzed sample. While the traditional EPV threshold of 10 was initially met (73 events/7 predictors = 10.4), we further validated the model’s stability by performing a sensitivity analysis with a reduced predictor set (4 predictors, EPV = 18.25) to align with the updated EPV ≥ 20 recommendation where feasible.

## Results

### Participant characteristics

The baseline characteristics of the development set were summarized according to their incident frailty status (Supplementary Table 5). Among the participants, the median age was 68.00 years (interquartile range: 64.00–72.00), and 678 individuals (62.8%) were women. After 1 year of follow-up, 73 older adults (6.8%) who transitioned into physical frailty exhibited baseline factors such as advanced age, increased risk of malnutrition, coughing while drinking, prefrailty, one-month fall occurrences, cognitive impairment, prolonged 5-time chair stand test duration, and reduced calf and mid-arm circumference compared to the non-frailty group (Supplementary Table 5).

The baseline characteristics of the temporal validation cohort closely mirrored those observed in the development cohort, albeit with a reduced proportion of female participants. Additionally, the temporal validation group exhibited a higher prevalence of reported histories of visual and hearing impairment, depression, hypertension, stroke, osteoarthrosis, prefrailty, dissatisfaction with social support, and limitations in activities of daily living (Supplementary Table 6). During the 12-month follow-up period, frailty manifested in 74 participants. Considering that a subset of participants did not complete the follow-up examinations in the temporal validation cohort, we conducted a comparative analysis of baseline characteristics for those with and without follow-up data. Our analysis revealed that participants lost to follow-up exhibited significantly higher rates of muscle loss, lower anthropometric values, and a greater prevalence of multiple chronic diseases, as detailed in Supplementary Table 4.

### Model development

**Figure 1:**
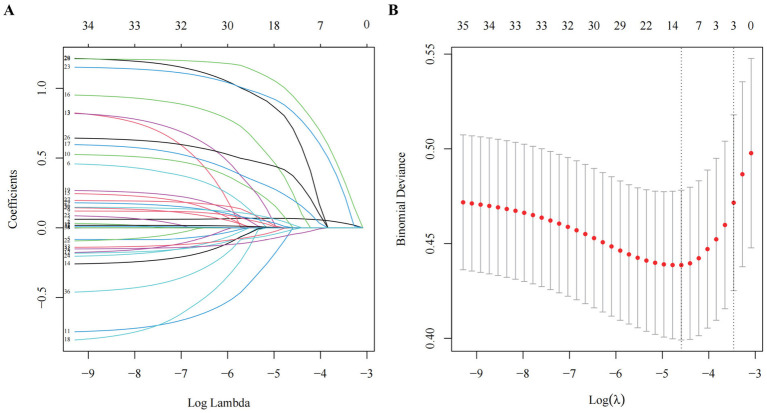
Screening of variables based on Lasso regression. **(A)** The variation characteristics of the coefficient of variables; **(B)** the selection process of the optimum value of the parameter *λ* in the Lasso regression model by cross-validation method.

**Figure 2:**
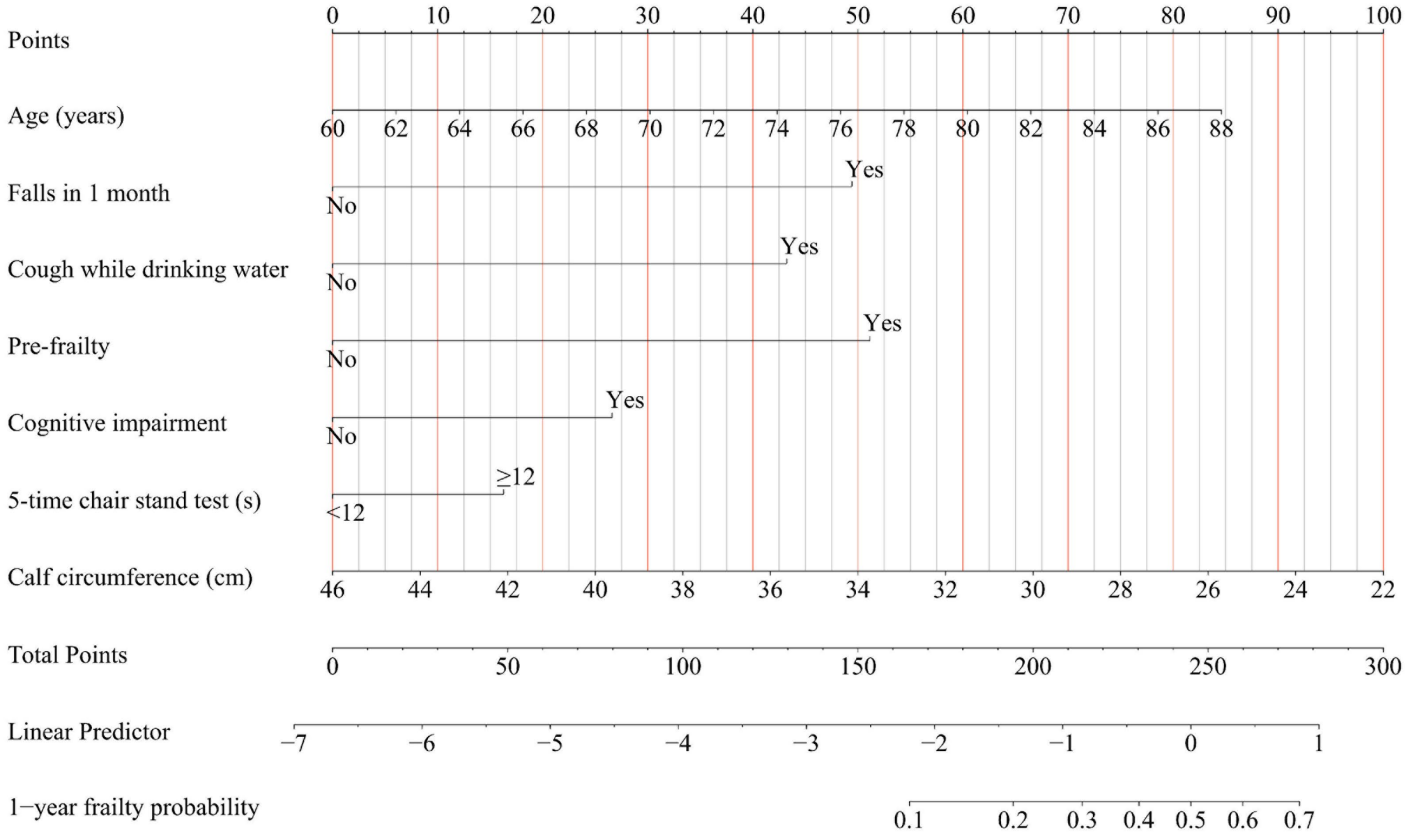
Nomogram for predicting the 1-year absolute risk of developing frailty in community-dwelling older adults. Cough while drinking water, Participants were inquired about whether they experienced coughing while drinking water; Falls in 1 month, Participants were queried regarding any incidents of falling within the preceding month.

**Figure 3:**
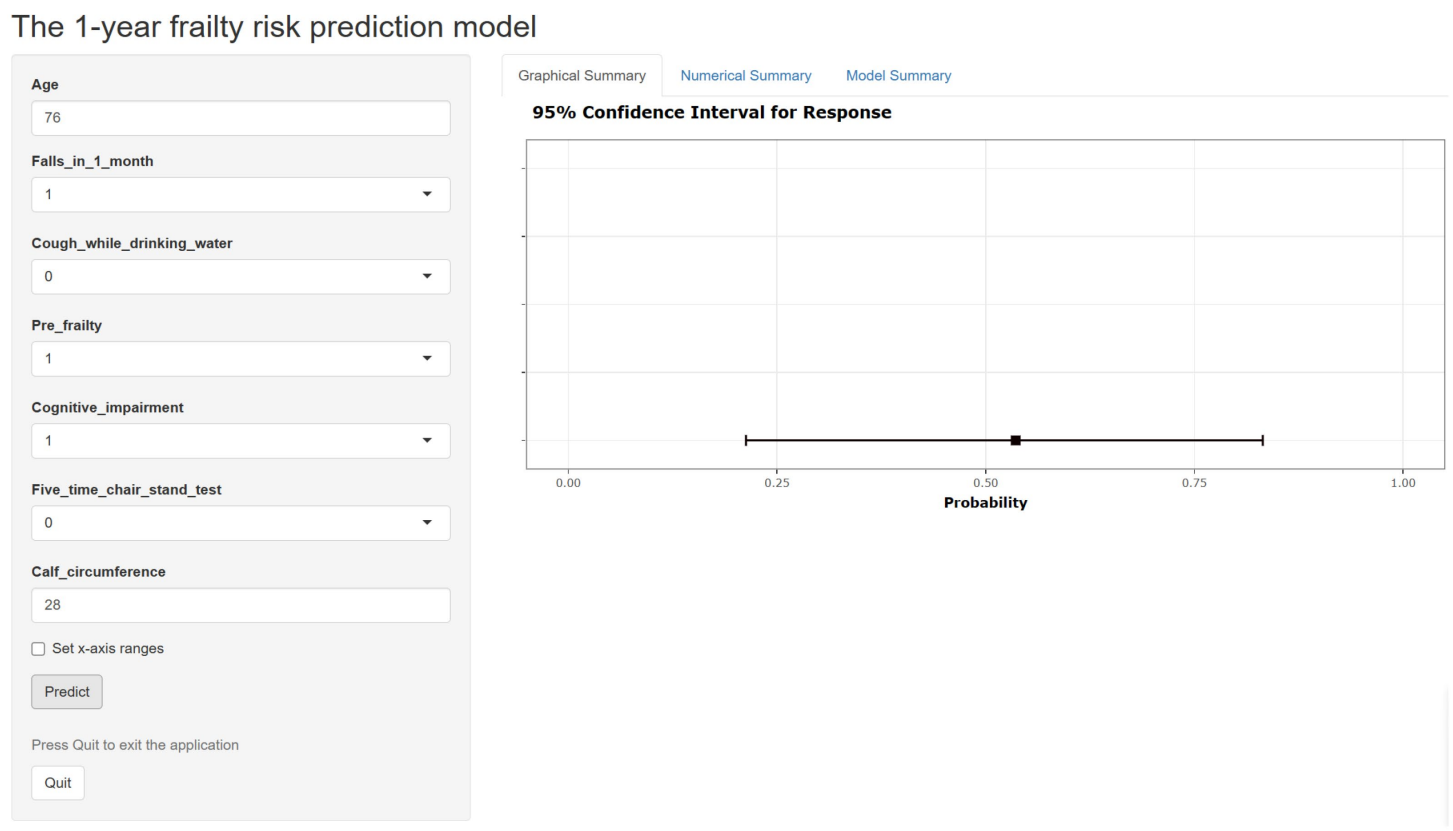
The web-based calculator for estimating the risk of frailty among community older adults (https://frailtyriskprediction.shinyapps.io/dynnomapp/).

In the development cohort study, the Lasso regression analysis was performed to screen the key variables (Figure 1). The Lasso technique subsequently identified seven predictors, which were utilized to construct the PRE-FRE risk prediction model. These predictors included age, occurrences of falls within 1 month, coughing while drinking water, pre-frailty, cognitive impairment, 5-time chair stand test, and calf circumference (Table 1; Supplementary Figure 2). The precise regression formula for the PRE-FRA model can be found in Supplementary Table 7. Utilizing these risk factors, we meticulously designed a nomogram to forecast the 1-year frailty probability among community-dwelling older adults (Figure 2). To enhance the accessibility and practicality of the PRE-FRA model, we created a user-friendly web-based calculator for automatic 1-year frailty risk assessment (Figure 3; available at: https://frailtyriskprediction.shinyapps.io/dynnomapp/).

**Table 1:** Logistic analyses of risk factors for incident frailty in the development cohort.

Predictors	Regression coefficient	Odds ratio (95% CI)	*p* value
Age, year	0.08	1.09 (1.04, 1.13)	<0.001
Falls in 1 month			0.022
No	Reference	Reference	
Yes	1.35	3.85 (1.10, 11.6)	
Cough while drinking water			<0.001
No	Reference	Reference	
Yes	1.18	3.25 (1.94, 5.46)	
Pre-frailty			<0.001
No	Reference	Reference	
Yes	1.40	4.04 (2.14, 8.33)	
Cognitive impairment			0.018
No	Reference	Reference	
Yes	0.73	2.07 (1.11, 3.72)	
5-time chair stand test (s)			0.099
<12	Reference	Reference	
≥12	0.44	1.56 (0.92, 2.65)	
Calf circumference (cm)	−0.11	0.89 (0.81, 0.98)	0.015
Intercept	−6.41	NA	NA

CI, confidence interval.

### Model performance and model validation

**Figure 4:**
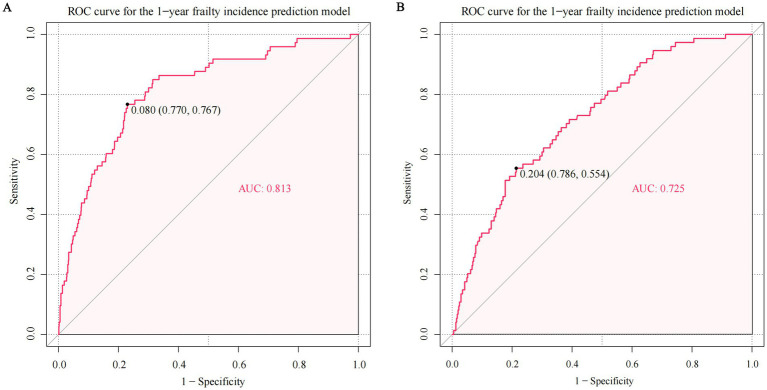
The area under the receiver-operator characteristic curve (AUROC) of predicting frailty in the development and temporal validation cohort. The ROC curve in the **(A)** development cohort and **(B)** temporal validation cohort. The AUROC for predicting frailty was 0.813 in the development cohort (95% confidence interval [CI] 0.761–0.865, *p* < 0.001) and 0.725 in the temporal validation cohort (95% CI 0.665–0.785, *p* < 0.001).

Figure 4 illustrates the AUROC curves for both the development cohort (AUROC = 0.81, 95% CI 0.76–0.87, *p* < 0.001) and the temporal validation cohort (AUROC = 0.73, 95% CI 0.67–0.79, *p* < 0.001). The clear separation of curves underscores the model’s ability to distinguish between frail and non-frailty individuals. The internal validation revealed minimal optimism, evidenced by an adjusted AUROC of 0.80, which underscores the model’s stable and consistent discriminative performance. Supplementary Figure 3 shows the calibration plots for both the development and temporal validation cohorts. The calibration slope of 0.93 and intercept of-0.14 in the development cohort indicate excellent calibration performance. Although there was a minor decrease in the temporal validation cohort (slope = 0.77, intercept = −0.34), the model still demonstrated acceptable calibration.

Supplementary Figure 4 presents the DCA, which demonstrates favorable net benefits across a wide spectrum of threshold risk values (4 to 40%) in both cohorts. This analysis underscores the model’s clinical utility by showing that it consistently outperforms the approach of managing all participants assuming either an increased or unaltered risk of frailty onset within the specified threshold probability range.

The diagnostic performance analysis across different risk thresholds revealed distinct clinical utilities. Lower thresholds demonstrated excellent efficacy in frailty exclusion, with a 2.5% cut-off achieving high sensitivity (0.92, 95% CI 0.83–0.97) and a favorable negative likelihood ratio (0.19, 95% CI 0.09–0.42). Conversely, higher thresholds ≥10% showed strong predictive value for frailty identification. Specifically, the 15% risk threshold yielded robust specificity (0.89, 95% CI 0.87–0.91) accompanied by a clinically meaningful positive likelihood ratio (4.68, 95% CI 3.51–6.24), as detailed in Supplementary Table 8.

### Sensitivity analysis

The AUROC for the complete case subset was 0.81, which is consistent with the main analysis (AUROC 0.81 in development cohort) (Supplementary Figure 5). This quantitative result supports the robustness of the model to missing data imputations. Supplementary Figure 6 present the AUROC values obtained using a reduced predictor set (age, pre-frailty, cough while drinking water, and calf circumference; EPV = 18.25). These analyses demonstrate that the model’s predictive performance remains robust and comparable to the overall analyzed sample, supporting the stability of our findings.

## Discussion

The primary objective was to develop and validate a 1-year incident frailty prediction model for older adults without baseline frailty residing in the community. Key predictors identified in our model encompassed age, falls in 1 month, coughing while drinking water, pre-frailty status, cognitive impairment, 5-time chair stand test, and calf circumference. Integrating these factors not only enhanced the model’s accuracy but also yielded valuable insights into the multifaceted nature of frailty in older adults. Rigorous internal and temporal validation processes substantiated the nomogram’s robust performance, showcasing exceptional discrimination and calibration abilities, thus reinforcing its reliability in predicting frailty risk.

Our PRE-FRA model advances existing frailty prediction tools by addressing key limitations in prior studies. Unlike cross-sectional models (11–16), our longitudinal design captures dynamic frailty progression, improving accuracy. Unlike Wu et al.’s retrospective approach (21), which risked circularity by using frailty index components as predictors, our prospective design ensures unbiased associations. Additionally, while several studies have recognized the potential benefits of prospective cohorts in frailty prediction (17–19), only a study has conducted external validation (19). Compared to Abe et al.’s model (AUROC: 0.71–0.72) (17), which lacked validation and baseline stratification, our PRE-FRA model achieved superior discrimination (AUROC: 0.81) and rigorously distinguished non-frailty from pre-frailty individuals. While Liu et al. (19) also employed temporal validation, their model’s lower AUROC (0.70) and omission of physical performance metrics (e.g., chair stand test) limited clinical utility. In comparison to the frailty prediction model developed by Liu Q et al. (19), our study unveils a notably superior AUROC for our prediction model in both the development cohort and the temporal validation cohort. The positive outcomes suggest that our PRE-FRA model holds promise for accurately predicting frailty across different contexts.

Our inclusion of objective measures (calf circumference, 5-time chair stand test) and novel predictors (coughing while drinking, recent falls) aligns with emerging evidence (46–50). and enhances predictive power. Calf circumference is a well-validated proxy for sarcopenia and muscle loss, which are central to frailty pathophysiology (46, 47). Reduced calf circumference has been associated with mobility decline and frailty in prior studies (46, 51), aligning with our findings. Incorporating performance-based assessments, such as the 5-time chair stand test, represents a shift toward objective measurements in frailty prediction, thereby enhancing prognostic precision. A 5-time chair stand test duration ≥12 s indicates diminished lower limb strength and functional capacity in older adults (48), aligning with frailty characteristics such as decreased mobility and independence. Coughing while drinking water - a distinctive predictor in our PRE-FRA model-has been understudied in prior research, underscoring the value of integrating unconventional indicators for frailty assessment. This symptom may reflect subclinical dysphagia, a well-established risk factor for malnutrition and frailty (49). Impaired swallowing can contribute to decreased caloric intake (52), recurrent respiratory infections (53, 54), and functional decline (55), all of which may accelerate frailty progression. Although seldom incorporated into existing frailty models, its inclusion in our framework is supported by longitudinal evidence linking dysphagia to worsening frailty. These refinements position PRE-FRA as a more comprehensive and generalizable tool. In addition, advanced age (17–19), pre-frailty (18), and cognitive impairment (19) emerged as critical factors associated with increased frailty in the previous frailty prediction model. The convergence of these predictors across studies validates their importance and underscores their relevance in various contexts. Notably, the significant association between cognitive function and frailty underscores the importance of assessing cognitive status when evaluating frailty risk, as cognitive impairment may exacerbate frailty-related decline and impact an individual’s ability to adhere to preventive interventions (56). Falls in the past month emerged as another significant predictor in our PRE-FRA model. Previous studies have demonstrated that frailty effectively predicts falls in older adults residing in the community (57, 58). Nonetheless, no prior prospective cohort investigation has validated that falls elevate the vulnerability to frailty. Our findings underscore the importance of falls not only as a consequence of frailty but also as a predictive factor, reinforcing the need for fall prevention strategies among older adults. The potential mechanism may be that the psychological repercussions of falls can lead to decreased confidence in one’s ability to perform daily activities, further reducing activity levels and exacerbating frailty development (50). The incorporation of this novel predictor showcases our PRE-FRA model’s capacity to capture nuanced aspects of health status that might be overlooked in traditional frailty assessments. Our findings corroborate and extend previous research on frailty prediction models. While our PRE-FRA model shares commonalities with existing literature, the integration of diverse predictors underscores our attempt to capture the multifaceted nature of frailty. This approach reflects the complexity of frailty development, which is influenced by a variety of physiological, functional, and cognitive factors.

The PRE-FRA model’s high sensitivity (0.92) for frailty exclusion at a 2.5% threshold and specificity (0.89) for frailty identification at a 15% threshold make it a valuable tool for early intervention. Healthcare providers can utilize this model to identify older adults at risk of frailty early on, allowing for targeted interventions to prevent or mitigate its progression. The net benefit improvement within the 4–40% predicted risk threshold indicates that the model can effectively guide clinical decisions. For instance, in community screening, individuals with estimated frailty risks in this range can be prioritized for preventive measures, optimizing resource allocation and reducing overtreatment. Such interventions may involve personalized exercise programs, nutritional counseling, cognitive training, and social engagement initiatives. Moreover, investigating the economic implications of implementing frailty prediction and prevention strategies could provide valuable insights for healthcare resource allocation. The PRE-FRA model demonstrates practical potential for integration into diverse community healthcare services, including community primary screening programs, older adult health management, and family physician assessments. Given its simplicity and the availability of a web-based calculator, it may also be adapted into mobile applications or public health toolkits to facilitate large-scale frailty risk assessment.

## Strengths and limitations

Our study presents several key strengths. The PRE-FRA model is specifically tailored to a diverse older adult population in Western China, addressing an underrepresented demographic in frailty research. It employs simple, easy-to-measure clinical and functional indicators that are feasible for use in community settings, and incorporates objective assessments to enhance predictive accuracy. The model’s robustness is supported by both internal and temporal validation, confirming its reliability across different time points. Furthermore, the development of a user-friendly, web-based calculator enhances its accessibility and facilitates practical implementation. These features collectively highlight the model’s potential for adaptation and replication in broader healthcare contexts.

Despite these strengths, our study has some limitations. First, our study’s specific setting in West China indeed presents certain limitations regarding the broader applicability of the PRE-FRA model. The unique demographic, cultural, and environmental factors of this region may affect the generalizability of our findings to other populations. However, this specificity also provides unique insights into frailty prediction in a previously underrepresented group, contributing valuable data to the field. Despite these limitations, our model offers a solid foundation for future research and can be adapted and validated in diverse settings. This work underscores the importance of region-specific studies in enhancing the robustness and inclusivity of frailty prediction models. Second, although we endeavored to include as many frailty-associated risk factors in our model as possible, there remains the possibility of unidentified variables for predicting frailty. Consequently, the application of the PRE-FRE model should be approached with caution to account for potential selection bias. Third, the bias-corrected frailty probability seems to be overestimated in the higher range. Limited sample size may lead to less reliable estimates, particularly in the higher range of frailty probabilities where fewer data points are available. This can cause the model to overfit the available data, resulting in an overestimation of frailty probabilities. While the model’s EPV of 10.4 met traditional criteria, contemporary guidelines advocate for ≥20. Our sensitivity analysis with fewer predictors (EPV = 18.25) mitigated this concern, but larger cohorts are needed for optimal EPV compliance. Fourth, the model showed overestimation at high predicted frailty probabilities (>35%), likely due to a small number of high-risk cases; future studies should address this through larger samples or improved calibration techniques. Finally, the model showed overestimation at high predicted frailty probabilities (>35%), likely due to a small number of high-risk cases; future studies should address this through larger samples or improved calibration techniques.

## Conclusion

This study developed and validated the PRE-FRA model, a novel tool for predicting 1-year frailty onset in initially non-frailty/pre-frailty older adults. Its innovations include: (1) integrating understudied predictors (e.g., calf circumference, coughing while drinking) with traditional risk factors; (2) employing longitudinal data from underrepresented regions (Western China); and (3) providing a user-friendly web-based calculator for real-time risk stratification. The model demonstrated high reliability, with robust discrimination and calibration across cohorts. Its clinical potential lies in identifying high-risk individuals for early intervention. While further external validation is warranted, the PRE-FRA model addresses critical gaps in frailty prediction by combining accessibility, multidomain assessment, and dynamic risk monitoring—aligning with geriatric care priorities in resource-limited settings.

## Data Availability

The raw data supporting the conclusions of this article will be made available by the authors, without undue reservation.
