# Supplementary material for: Development and validation of PRE-FRA (PREdiction of FRAilty risk in community older adults) frailty prediction model
Source: Front Public Health. 2025 Jun 27;13:1593668. doi: 10.3389/fpubh.2025.1593668 (PMC12245915; doi:10.3389/fpubh.2025.1593668)
Supplement: Supplementary file 1 [file Supplementary_file_1.docx]

**Supplementary Table 1**

Health variables and cut-points for frailty

| **Items** | **Description** | **Cut point** |
| --- | --- | --- |
| Shrinking | Unintentional weight loss: >4.5 kg or >5% of body weight in past 12 months | Yes = 1, No = 0 |
| Weakness | Handgrip strength was measured in kilograms using CAMRY EH101 dynamometers. Low handgrip strength (LGS) was defined by gender and BMI-specific cutoffs: for women, ≤17 kg (BMI ≤23), ≤17.3 kg (BMI 23.1–26), ≤18 kg (BMI 26.1–29), and ≤21 kg (BMI >29); for men, ≤29 kg (BMI ≤24), ≤30 kg (BMI 24.1–28), and ≤32 kg (BMI >28). | Yes = 1, No = 0 |
| Exhaustion | Self-reported exhaustion was characterized as feelings of exertion in which tasks required considerable effort or the patient experienced an inability to initiate tasks for more than three consecutive days within the past week. | Yes = 1, No = 0 |
| Slowness | Low gait speed during a 4.57-meter walk was determined as exceeding 6 seconds for men taller than 173 cm or women taller than 159 cm, and surpassing 7 seconds for men equal to or shorter than 173 cm or women equal to or shorter than 159 cm. | Yes = 1, No = 0 |
| Inactivity | Low physical activity was defined as <383 kcal/week for men and <270 kcal/week for women. | Yes = 1, No = 0 |

**Supplementary Table 2**

Candidate predictors

| **Candidate predictor**  **variable** | **Description** | **Category** | **Units/Categories** |
| --- | --- | --- | --- |
| Age | Validated age based on self-reported | Dichotomized | 0.60-69 years  1.70-79 years  2.≥80 years |
| Sex | Self-reported | Binary |  |
| Marital status | Current marital status | Binary | 0.unmarried/widowed  1.married |
| Education | No education (0 years), Primary (1-6 years), Secondary (6-9 years), Higher (≥9 years) | Dichotomized | 0.No education  1.Primary  2.Secondary  3.More than secondary |
| Social support | The Social Support Rating Scale (SSRS) was employed to evaluate individual levels of social support^1^. Scores range from 0 to 66 (higher = greater support), with <30 indicating low support and ≥30 indicating high support. | Binary | 0.low level  1.high level |
| Activity of daily living (ADL) limitation | ADL limitation was defined as experiencing difficulty or requiring assistance with at least one of six daily activities, including bathing, dressing, indoor transferring, toileting, eating, and continence | Binary | 0. yes  1. no |
| Sleep disorder | The Pittsburgh Sleep Quality Index (PSQI) assessed sleep quality, categorizing participants into two groups: poor sleep (PSQI ≥ 11) and good sleep (PSQI < 11)^6^. | Binary | 0. good sleep quality  1. sleep disorder |
| Sleep duration | Participants were queried about their nightly sleep duration over the past month. | Continuous | hour |
| Visual impairment | The participants were queried about their ability to perceive small, proximate objects, such as books and newspapers | Binary | 0. yes  1. no |
| Hearing impairment | Participants were queried if they could hear clearly  under normal volume and speed conditions. | Binary | 0. yes  1. no |
| Pain | Participants reported any pain lasting ≥1 month in the six months before the study. Those with such pain were classified as having chronic pain; others, as pain-free. | Binary | 0. had no pain  1. pain |
| Malnutrition risk | The Mini Nutritional Assessment Short Form (MNA-SF) was used to assess participants' nutritional status^5^. This 6-item tool, designed for older adults, evaluates nutritional and health-related factors. Based on MNA-SF scores, participants were classified as: “normal nutritional status” (12-14), “at risk of malnutrition” (8-11), or “malnourished” (<7). | Binary | 0. normal nutritional status  1. risk of malnutrition/ malnourished |
| Cough while drinking water | Participants were inquired about whether they experienced coughing while drinking water. | Binary | 0. no  1. yes |
| Frailty status | Frailty was assessed using Fried's five phenotype criteria^7^. Participants scoring 3-5 were classified as frail, 1-2 as pre-frail, and 0 as non-frail. | Binary | 0. non-frailty  1. pre-frailty |
| Falls in 1 month | Participants were queried regarding any incidents of falling within the preceding month. | Binary | 0. no  1. yes |
| 5-time chair stand test | Physical functioning was measured via a 5-time chair stand test (armless chair, 0.47 m height)^8^. Time (seconds) to complete five stands without hand use was recorded; ≥12 seconds indicated low performance. | Binary | 0. <12 seconds  1. ≥12 seconds |
| Skeletal mass index | Appendicular skeletal muscle mass was measured via bioimpedance analysis (InBody 770). The skeletal muscle index (kg/m²) was calculated as appendicular muscle mass (kg) divided by height squared (m²). | Binary | kg/m^2^ |
| Body mass index | The body mass index (BMI) was computed by dividing the weight (in kilograms) by the square of the height (in meters). | Continuous | kg/m^2^ |
| Calf circumference | The subject stands with their feet positioned shoulder-width apart, while the tester stands beside them. A ruler was positioned horizontally, passing through the thickest part of the gastrocnemius muscle (located in the calf), in order to measure its circumference. The average of the two measurements was considered as the recorded value. | Continuous | cm |
| Waist circumference | Place the tape snugly at the midpoint between the lower ribs and iliac crest. Measure waist circumference after normal exhalation, then average two measurements for the final value. | Continuous | cm |
| Mid-arm circumference | Measure the circumference of the upper arm at its midpoint. Calculate the average value of the two measurements to determine the final measurement. | Continuous | cm |
| Triceps skinfold | Pinch the triceps skinfold between your thumb and index finger, avoiding muscle tissue. Lift the fold, place the caliper 1 cm below your fingers, perpendicular to its axis. Squeeze until the jaws align and the reading stabilizes. Record the average of two measurements. | Continuous | mm |
| Hip circumference | Hip circumference was measured horizontally at the level of the greatest gluteal protrusion using a flexible tape, parallel to the floor and snug but non-compressive. The average of two measurements was recorded. | Continuous | cm |
| Cognitive impairment | Cognitive function was assessed using the Short Portable Mental Status Questionnaire (SPMSQ)^4^, with impairment thresholds adjusted for education level: (1) <6 years, score <7; (2) 6–9 years, score <8; (3) >9 years, score <9. | Binary | 0. normal cognition  1. cognitive impairment |
| Depression | Depressive symptoms were assessed using the 15-item Geriatric Depression Scale (GDS-15; range 0-15), with scores ≥5 indicating clinically significant symptoms^2^. | Binary | 0. no depressive symptom  1. depressive symptom |
| Anxiety | Anxiety symptoms were assessed utilizing the 7-item Generalized Anxiety Disorder Scale (GAD-7)^3^. The total score on the GAD-7 scale ranges from 0 to 21, with a score of 5 points or higher indicating the presence of anxiety symptoms. | Binary | 0. no anxiety symptom  1. anxiety symptom |
| Hypertension | The participants were asked whether a doctor had diagnosed them with hypertension. | Binary | 0. no  1. yes |
| Coronary heart disease | The participants were asked whether a doctor had diagnosed them with coronary heart disease. | Binary | 0. no  1. yes |
| Stroke | The participants were asked whether a doctor had diagnosed them with stroke. | Binary | 0. no  1. yes |
| Diabetes mellitus | The participants were asked whether a doctor had diagnosed them with diabetes mellitus. | Binary | 0. no  1. yes |
| COPD | The participants were asked whether a doctor had diagnosed them with COPD. | Binary | 0. no  1. yes |
| Osteoarthrosis | The participants were asked whether a doctor had diagnosed them with osteoarthrosis. | Binary | 0. no  1. yes |
| Cancer | The participants were asked whether a doctor had diagnosed them with cancer. | Binary | 0. no  1. yes |

COPD, chronic obstructive pulmonary disease.

**Supplementary Table 3**

The numbers (percentage) of the missing variables

| **Characteristics *** | **Number (%) with missing data** | |
| --- | --- | --- |
|  | **Development** **cohort (n = 1079)** | **Temporal validation cohort(n = 481)** |
| Education | 15 (1.4) | 0 |
| Hearing impairment | 0 | 1 (0.2) |
| Cough while drinking water | 0 | 1 (0.2) |
| Malnutrition risk | 0 | 4 (0.8) |
| 5-time chair stand test | 4 (0.4) | 15 (3.1) |
| Skeletal mass index | 67 (6.2) | 0 |
| Body mass index | 0 | 4 (0.8) |
| Pain | 0 | 1 (0.2) |
| Cognitive function | 4 (0.4) | 3 (0.6) |
| Depression | 0 | 4 (0.8) |
| Anxiety | 1 (0.1) | 3 (0.6) |

* List only the variables with missing data. Missing data were handled using multiple imputation.

**Supplementary Table 4**

Characteristics of participants who were lost to follow-up and returned

| **Characteristics** | **Development cohort** | | | **Temporal validation cohort** | | |
| --- | --- | --- | --- | --- | --- | --- |
|  | **Returned**  **(n=1079)** | **Lost to follow-up (n=390)** | **P value** | **Returned** | **Lost to follow-up (n=1320)** | **P value** |
|  |  |  |  | (n=481) |  |  |
| Age (year), median (IQR) | 68.00 (64.00, 72.00) | 68.00 (64.00, 73.00) | 0.592 | 67.00 (64.00, 71.00) | 66.00 (63.00, 72.00) | 0.068 |
| Female, no. (%) | 678 (62.8) | 238 (61.0) | 0.568 | 252 (52.4) | 794 (60.2) | 0.004 |
| Education, no. (%) |  |  | 0.050 |  |  | <0.001 |
| No education | 348 (32.3) | 152 (39.0) |  | 149 (31.0) | 536 (40.6) |  |
| Primary | 419 (38.8) | 127 (32.6) |  | 200 (41.6) | 473 (35.8) |  |
| Secondary | 191 (17.7) | 62 (15.9) |  | 84 (17.5) | 158 (12.0) |  |
| More than secondary | 121 (11.2) | 49 (12.6) |  | 48 (10.0) | 153 (11.6) |  |
| Married, no. (%) | 901 (83.5) | 314 (80.5) | 0.208 | 387 (80.5) | 1024 (77.6) | 0.212 |
| Social support, no. (%) |  |  | 0.095 |  |  | 0.158 |
| Satisfaction | 1052 (97.5) | 373 (95.6) |  | 14 (2.9) | 60 (4.5) |  |
| Dissatisfaction | 27 (2.5) | 17 (4.4) |  | 467 (97.1) | 1260 (95.5) |  |
| ADL limitation, no. (%) | 44 ( 4.1) | 18 ( 4.6) | 0.76 | 39 ( 8.1) | 135 (10.2) | 0.209 |
| Sleep disorder, no. (%) | 125 (11.6) | 56 (14.4) | 0.181 | 55 (11.4) | 181 (13.7) | 0.235 |
| Sleep duration (hours), no. (%) | 7.00 (6.05, 8.00) | 7.50 (6.50, 8.20) | 0.013 | 8.00 (7.00, 8.50) | 8.00 (7.00, 9.00) | 0.984 |
| Visual impairment, no. (%) | 547 (50.7) | 206 (52.8) | 0.509 | 306 (63.6) | 889 (67.3) | 0.154 |
| Hearing impairment, no. (%) | 355 (32.9) | 158 (40.5) | 0.008 | 196 (40.7) | 543 (41.1) | 0.925 |
| Pain, no. (%) | 333 (30.9) | 122 (31.3) | 0.928 | 184 (38.3) | 591 (44.8) | 0.016 |
| Malnutrition risk, no. (%) | 151 (14.0) | 95 (24.4) | <0.001 | 82 (17.0) | 276 (20.9) | 0.080 |
| Cough while drinking water, no. (%) | 277 (25.7) | 99 (25.4) | 0.965 | 121 (25.2) | 306 (23.2) | 0.419 |
| Frailty status, no. (%) |  |  | <0.001 |  |  | <0.001 |
| Non-frailty | 512 (47.5) | 145 (37.2) |  | 87 (18.1) | 157 (11.9) |  |
| Pre-frailty | 567 (52.5) | 217 (55.6) |  | 394 (81.9) | 790 (59.8) |  |
| Frailty | 0 | 28(7.2) |  | 0 | 373 (28.3) |  |
| Falls in 1 month, no. (%) | 24 (2.2) | 12 (3.1) | 0.458 | 14 (2.9) | 48 (3.6) | 0.548 |
| 5-time chair stand test (s), median (IQR) | 11.17 (9.38, 13.15) | 11.78 (9.82, 13.83) | 0.001 | 11.39 (9.67, 13.37) | 12.11 (10.07, 14.29) | <0.001 |
| 5-time chair stand test (s), no. (%) |  |  | 0.003 |  |  | <0.001 |
| <12 | 677 (62.7) | 210 (53.8) |  | 281 (58.4) | 641 (48.6) |  |
| ≥12 | 402 (37.3) | 180 (46.2) |  | 200 (41.6) | 679 (51.4) |  |
| Skeletal mass index (kg/m^2^), median (IQR) | 6.50 (5.90, 7.10) | 6.40 (5.80, 7.10) | 0.105 | 6.60 (6.00, 7.30) | 6.40 (5.80, 7.10) | <0.001 |
| Body mass index (kg/m^2^), median (IQR) | 25.14 (22.93, 27.49) | 24.61 (22.36, 27.08) | 0.007 | 24.87 (22.89, 27.30) | 24.67 (22.10, 27.06) | 0.103 |
| Calf circumference (cm), median (IQR) | 34.03 (32.10, 35.75) | 33.50 (31.26, 35.75) | 0.004 | 34.75 (32.65, 36.95) | 34.10 (31.99, 36.30) | <0.001 |
| Waist circumference (cm), median (IQR) | 86.25 (79.75, 93.22) | 85.53 (77.85, 92.75) | 0.166 | 87.80 (81.00, 94.10) | 86.00 (78.35, 94.00) | 0.006 |
| Mid-arm circumference (cm), median (IQR) | 27.55 (25.73, 29.33) | 27.25 (24.96, 28.99) | 0.002 | 28.50 (26.45, 30.70) | 28.05 (25.90, 30.30) | 0.012 |
| Triceps skinfold (mm), median (IQR) | 16.50 (11.00, 23.12) | 16.02 (10.75, 23.11) | 0.583 | 22.00 (16.55, 27.45) | 22.25 (16.50, 28.05) | 0.583 |
| Hip circumference (cm), median (IQR) | 94.35 (90.45, 98.50) | 93.75 (89.18, 98.57) | 0.139 | 95.70 (91.00, 100.00) | 94.00 (89.88, 99.00) | 0.006 |
| Cognitive impairment, no. (%) | 159 (14.7) | 80 (20.5) | 0.010 | 55 (11.4) | 238 (18.0) | 0.001 |
| Depression, no. (%) | 17 (1.6) | 30 (7.7) | <0.001 | 17 (3.5) | 67 (5.1) | 0.213 |
| Anxiety, no. (%) | 142 (13.2) | 54 (13.8) | 0.799 | 82 (17.0) | 262 (19.8) | 0.204 |
| Hypertension, no. (%) | 391 (36.2) | 128 (32.8) | 0.251 | 140 (29.1) | 385 (29.2) | 1.000 |
| Coronary heart disease, no. (%) | 54 (5.0) | 29 (7.4) | 0.098 | 15 (3.1) | 69 (5.2) | 0.080 |
| Stroke, no. (%) | 55 (5.1) | 17 (4.4) | 0.658 | 9 (1.9) | 30 (2.3) | 0.738 |
| Diabetes mellitus, no. (%) | 126 (11.7) | 43 (11.0) | 0.800 | 41 (8.5) | 108 (8.2) | 0.891 |
| COPD, no. (%) | 91 (8.4) | 38 (9.7) | 0.497 | 8 (1.7) | 29 (2.2) | 0.604 |
| Osteoarthrosis, no. (%) | 298 (27.6) | 114 (29.2) | 0.588 | 40 (8.3) | 158 (12.0) | 0.036 |
| Cancer, no. (%) | 23 (2.1) | 5 (1.3) | 0.403 | 3 (0.6) | 8 (0.6) | 1.000 |

IQR, interquartile range; ADL, Activity of daily living; COPD, chronic obstructive pulmonary disease.

**Supplementary Table 5**

Baseline characteristics of participants according to incident frailty status.

| **Characteristics** | **All participants**  **(n = 1079)** | **Development cohort** | | | **P value** |
| --- | --- | --- | --- | --- | --- |
|  |  | **Non-frailty/pre-frailty**  **(n = 1006)** | | **Frailty**  **(n = 73)** |  |
| Age (years), median (IQR) | 68.00 (64.00, 72.00) | 67.00 (64.00, 71.00) | 72.00 (68.00, 77.00) | | <0.001 |
| Female, no. (%) | 678 (62.8) | 630 (62.6) | 48 (65.8) | | 0.683 |
| Education, no. (%) |  |  |  | | 0.823 |
| No education | 348 (32.3) | 323 (32.1) | 25 (34.2) | |  |
| Primary | 419 (38.8) | 390 (38.8) | 29 (39.7) | |  |
| Secondary | 191 (17.7) | 181 (18.0) | 10 (13.7) | |  |
| More than secondary | 121 (11.2) | 112 (11.1) | 9 (12.3) | |  |
| Married, no. (%) | 901 (83.5) | 844 (83.9) | 57 (78.1) | | 0.259 |
| Social support, no. (%) |  |  |  | | 0.601 |
| Satisfaction | 1052 (97.5) | 982 (97.6) | 70 (95.9) | |  |
| Dissatisfaction | 27 (2.5) | 24 (2.4) | 3 (4.1) | |  |
| ADL limitation, no. (%) | 44 ( 4.1) | 38 ( 3.8) | 6 ( 8.2) | | 0.122 |
| Sleep disorder, no. (%) | 125 (11.6) | 112 (11.1) | 13 (17.8) | | 0.126 |
| Sleep duration (hours), median (IQR) | 7.00 (6.05, 8.00) | 7.00 (6.00, 8.00) | 7.50 (6.50, 8.50) | | 0.276 |
| Visual impairment, no. (%) | 547 (50.7) | 503 (50.0) | 44 (60.3) | | 0.115 |
| Hearing impairment, no. (%) | 355 (32.9) | 324 (32.2) | 31 (42.5) | | 0.094 |
| Pain, no. (%) | 333 (30.9) | 308 (30.6) | 25 (34.2) | | 0.605 |
| Malnutrition risk, no. (%) | 151 (14.0) | 134 (13.3) | 17 (23.3) | | 0.028 |
| Cough while drinking water, no. (%) | 277 (25.7) | 240 (23.9) | 37 (50.7) | | <0.001 |
| Frailty status, no. (%) |  |  |  | | <0.001 |
| Non-frailty | 512 (47.5) | 501 (49.8) | 11 (15.1) | |  |
| Pre-frailty | 567 (52.5) | 505 (50.2) | 62 (84.9) | |  |
| Falls in 1 month, no. (%) | 24 ( 2.2) | 19 ( 1.9) | 5 ( 6.8) | | 0.018 |
| 5-time chair stand test (s), median (IQR) | 11.17 (9.38, 13.15) | 11.07 (9.31, 13.05) | 12.45 (10.60, 14.98) | | <0.001 |
| 5-time chair stand test (s), no. (%) |  |  |  | | 0.001 |
| <12 | 677 (62.7) | 645 (64.1) | 32 (43.8) | |  |
| ≥12 | 402 (37.3) | 361 (35.9) | 41 (56.2) | |  |
| Skeletal mass index (kg/m2), median (IQR) | 6.50 (5.90, 7.10) | 6.50 (5.90, 7.10) | 6.10 (5.60, 6.80) | | 0.004 |
| Body mass index (kg/m2), median (IQR) | 25.14 (22.93, 27.49) | 25.14 (22.93, 27.43) | 24.73 (23.13, 28.17) | | 0.727 |
| Calf circumference (cm), median (IQR) | 34.03 (32.10, 35.75) | 34.10 (32.15, 35.85) | 33.40 (31.35, 34.95) | | 0.006 |
| Waist circumference (cm), median (IQR) | 86.25 (79.75, 93.22) | 86.20 (79.71, 93.10) | 86.75 (81.30, 96.40) | | 0.391 |
| Mid-arm circumference (cm), median (IQR) | 27.55 (25.73, 29.33) | 27.60 (25.75, 29.45) | 27.15 (25.00, 28.75) | | 0.045 |
| Triceps skinfold (mm), median (IQR) | 16.50 (11.00, 23.12) | 16.50 (11.00, 23.24) | 16.75 (11.50, 21.50) | | 0.998 |
| Hip circumference (cm), median (IQR) | 94.35 (90.45, 98.50) | 94.45 (90.61, 98.50) | 93.55 (89.75, 99.10) | | 0.428 |
| Cognitive impairment, no. (%) | 159 (14.7) | 140 (13.9) | 19 (26.0) | | 0.008 |
| Depression, no. (%) | 17 (1.6) | 16 (1.6) | 1 (1.4) | | 1.000 |
| Anxiety, no. (%) | 142 (13.2) | 130 (12.9) | 12 (16.4) | | 0.497 |
| Hypertension, no. (%) | 391 (36.2) | 362 (36.0) | 29 (39.7) | | 0.606 |
| Coronary heart disease, no. (%) | 54 (5.0) | 49 (4.9) | 5 (6.8) | | 0.638 |
| Stroke, no. (%) | 55 (5.1) | 48 (4.8) | 7 (9.6) | | 0.126 |
| Diabetes mellitus, no. (%) | 126 (11.7) | 121 (12.0) | 5 (6.8) | | 0.254 |
| COPD, no. (%) | 91 (8.4) | 80 (8.0) | 11 (15.1) | | 0.058 |
| Osteoarthrosis, no. (%) | 298 (27.6) | 277 (27.5) | 21 (28.8) | | 0.927 |
| Cancer, no. (%) | 23 (2.1) | 20 (2.0) | 3 (4.1) | | 0.428 |

IQR, interquartile range; ADL, Activity of daily living; COPD, chronic obstructive pulmonary disease.

**Supplementary Table 6**

Participants’ characteristics in cohort studies

| **Characteristics** | **All participants**  **( n = 1560)** | **Development cohort**  **(n = 1079)** | **Temporal validation cohort**  **(n = 481)** | **P value** |
| --- | --- | --- | --- | --- |
| Age (years), median (IQR) | 67.00 (64.00, 72.00) | 68.00 (64.00, 72.00) | 67.00 (64.00, 71.00) | 0.097 |
| Female, no. (%) | 930 (59.6) | 678 (62.8) | 252 (52.4) | <0.001 |
| Married, no. (%) | 1288 (82.6) | 901 (83.5) | 387 (80.5) | 0.164 |
| Education, no. (%) |  |  |  | 0.733 |
| No education | 497 (31.9) | 348 (32.3) | 149 (31.0) |  |
| Primary | 619 (39.7) | 419 (38.8) | 200 (41.6) |  |
| Secondary | 275 (17.6) | 191 (17.7) | 84 (17.5) |  |
| More than secondary | 169 (10.8) | 121 (11.2) | 48 (10.0) |  |
| Social support, no. (%) |  |  |  |  |
| Satisfaction | 1066 (68.3) | 1052 (97.5) | 14 (2.9) | <0.001 |
| Dissatisfaction | 494 (31.7) | 27 (2.5) | 467 (97.1) |  |
| ADL limitation, no. (%) | 83 ( 5.3) | 44 ( 4.1) | 39 ( 8.1) | 0.002 |
| Sleep disorder, no. (%) | 180 (11.5) | 125 (11.6) | 55 (11.4) | 1.000 |
| Sleep duration (hours), median (IQR) | 7.50 (6.50, 8.00) | 7.00 (6.05, 8.00) | 8.00 (7.00, 8.50) | <0.001 |
| Visual impairment, no. (%) | 853 (54.7) | 547 (50.7) | 306 (63.6) | <0.001 |
| Hearing impairment, no. (%) | 551 (35.3) | 355 (32.9) | 196 (40.7) | 0.003 |
| Pain, no. (%) | 517 (33.1) | 333 (30.9) | 184 (38.3) | 0.005 |
| Malnutrition risk, no. (%) | 233 (14.9) | 151 (14.0) | 82 (17.0) | 0.137 |
| Cough while drinking water, no. (%) | 398 (25.5) | 277 (25.7) | 121 (25.2) | 0.878 |
| Frailty status, no. (%) |  |  |  | <0.001 |
| Non-frailty | 599 (38.4) | 512 (47.5) | 87 (18.1) |  |
| Pre-frailty | 961 (61.6) | 567 (52.5) | 394 (81.9) |  |
| Falls in 1 month, no. (%) | 38 ( 2.4) | 24 ( 2.2) | 14 ( 2.9) | 0.526 |
| 5-time chair stand test (s), median (IQR) | 11.25 (9.44, 13.23) | 11.17 (9.38, 13.15) | 11.39 (9.67, 13.37) | 0.079 |
| 5-time chair stand test (s), no. (%) |  |  |  | 0.118 |
| <12 | 958 (61.4) | 677 (62.7) | 281 (58.4) |  |
| ≥12 | 602 (38.6) | 402 (37.3) | 200 (41.6) |  |
| Skeletal mass index (kg/m^2^), median (IQR) | 6.50 (5.90, 7.10) | 6.50 (5.90, 7.10) | 6.60 (6.00, 7.30) | 0.002 |
| Body mass index (kg/m^2^), median (IQR) | 25.08 (22.92, 27.41) | 25.14 (22.93, 27.49) | 24.87 (22.89, 27.30) | 0.121 |
| Calf circumference (cm), median (IQR) | 34.20 (32.25, 36.06) | 34.03 (32.10, 35.75) | 34.75 (32.65, 36.95) | <0.001 |
| Waist circumference (cm), median (IQR) | 86.80 (80.10, 93.50) | 86.25 (79.75, 93.22) | 87.80 (81.00, 94.10) | 0.029 |
| Mid-arm circumference (cm), median (IQR) | 27.88 (25.85, 29.75) | 27.55 (25.73, 29.33) | 28.50 (26.45, 30.70) | <0.001 |
| Triceps skinfold (mm), median (IQR) | 18.85 (12.00, 24.76) | 16.50 (11.00, 23.12) | 22.00 (16.55, 27.45) | <0.001 |
| Hip circumference (cm), median (IQR) | 94.75 (90.65, 98.76) | 94.35 (90.45, 98.50) | 95.70 (91.00, 100.00) | 0.021 |
| Cognitive impairment, no. (%) | 214 (13.7) | 159 (14.7) | 55 (11.4) | 0.095 |
| Depression, no. (%) | 34 (2.2) | 17 (1.6) | 17 (3.5) | 0.024 |
| Anxiety, no. (%) | 224 (14.4) | 142 (13.2) | 82 (17.0) | 0.052 |
| Hypertension, no. (%) | 531 (34.0) | 391 (36.2) | 140 (29.1) | 0.007 |
| Coronary heart disease, no. (%) | 69 (4.4) | 54 (5.0) | 15 (3.1) | 0.124 |
| Stroke, no. (%) | 64 (4.1) | 55 (5.1) | 9 (1.9) | 0.005 |
| Diabetes mellitus, no. (%) | 167 (10.7) | 126 (11.7) | 41 (8.5) | 0.076 |
| COPD, no. (%) | 99 (6.3) | 91 (8.4) | 8 (1.7) | <0.001 |
| Osteoarthrosis, no. (%) | 338 (21.7) | 298 (27.6) | 40 (8.3) | <0.001 |
| Cancer, no. (%) | 26 (1.7) | 23 (2.1) | 3 (0.6) | 0.053 |

IQR, interquartile range; ADL, Activity of daily living; COPD, chronic obstructive pulmonary disease.

**Supplementary Table 7**

The formula for the frailty risk prediction model in the development cohort

| 1-year frailty risk (%)=1/(1 + exp ^ [ - ( - 6.41  + 0.08 for age (years)  + 0 for those who haven't fallen in 1 month or 1.35 for those who had fallen within 1 month  + 0 for those who drink water without coughing or 1.18 for those who cough while drinking water  + 0 for non-frailty or 1.40 for pre-frailty  + 0 for normal cognition or 0.73 for cognitive impairment  + 0 for 5-time chair stand test < 12s or 0.44 for 5-time chair stand test ≥ 12s  - 0.11 × calf circumference (cm) ]) |
| --- |

The scoring system’s intercept is expressed as -6.41; the other numbers represent the regression coefficients (weight) of each risk factor.

**Supplementary Table 8** Diagnostic performance measures at different risk thresholds of the final prediction model

| **Outcomes and risk thresholds** | **Sensitivity (95% CI)** | **Specificity (95% CI)** | **Predictive value (95% CI)** | | **Likelihood ratio (95% CI)** | |
| --- | --- | --- | --- | --- | --- | --- |
|  |  |  | **Positive** | **Negative** | **Positive** | **Negative** |
| ≥2.5% | 0.92 (0.83, 0.97) | 0.43 (0.39, 0.46) | 0.10 (0.08, 0.13) | 0.99 (0.97, 0.99) | 1.60 (1.46, 1.74) | 0.19 (0.09, 0.42) |
| ≥5% | 0.86 (0.76, 0.93) | 0.65 (0.62, 0.68) | 0.15 (0.12, 0.19) | 0.98 (0.97, 0.99) | 2.45 (2.16, 2.77) | 0.21 (0.12, 0.38) |
| ≥10% | 0.62 (0.50, 0.73) | 0.82 (0.79, 0.84) | 0.20 (0.15, 0.26) | 0.97 (0.95, 0.98) | 3.43 (2.74, 4.29) | 0.47 (0.35, 0.63) |
| ≥15% | 0.51 (0.39, 0.63) | 0.89 (0.87, 0.91) | 0.25 (0.19, 0.33) | 0.96 (0.95, 0.97) | 4.68 (3.51, 6.24) | 0.55 (0.44, 0.70) |
| ≥30% | 0.18 (0.10, 0.29) | 0.98 (0.97, 0.99) | 0.38 (0.22, 0.56) | 0.94 (0.93, 0.96) | 8.53 (4.46, 16.33) | 0.84 (0.75, 0.93) |
| ≥40% | 0.10 (0.04, 0.19) | 0.99 (0.99, 1.00) | 0.54 (0.25, 0.81) | 0.94 (0.92, 0.95) | 16.08 (5.55, 46.60) | 0.91 (0.84, 0.98) |


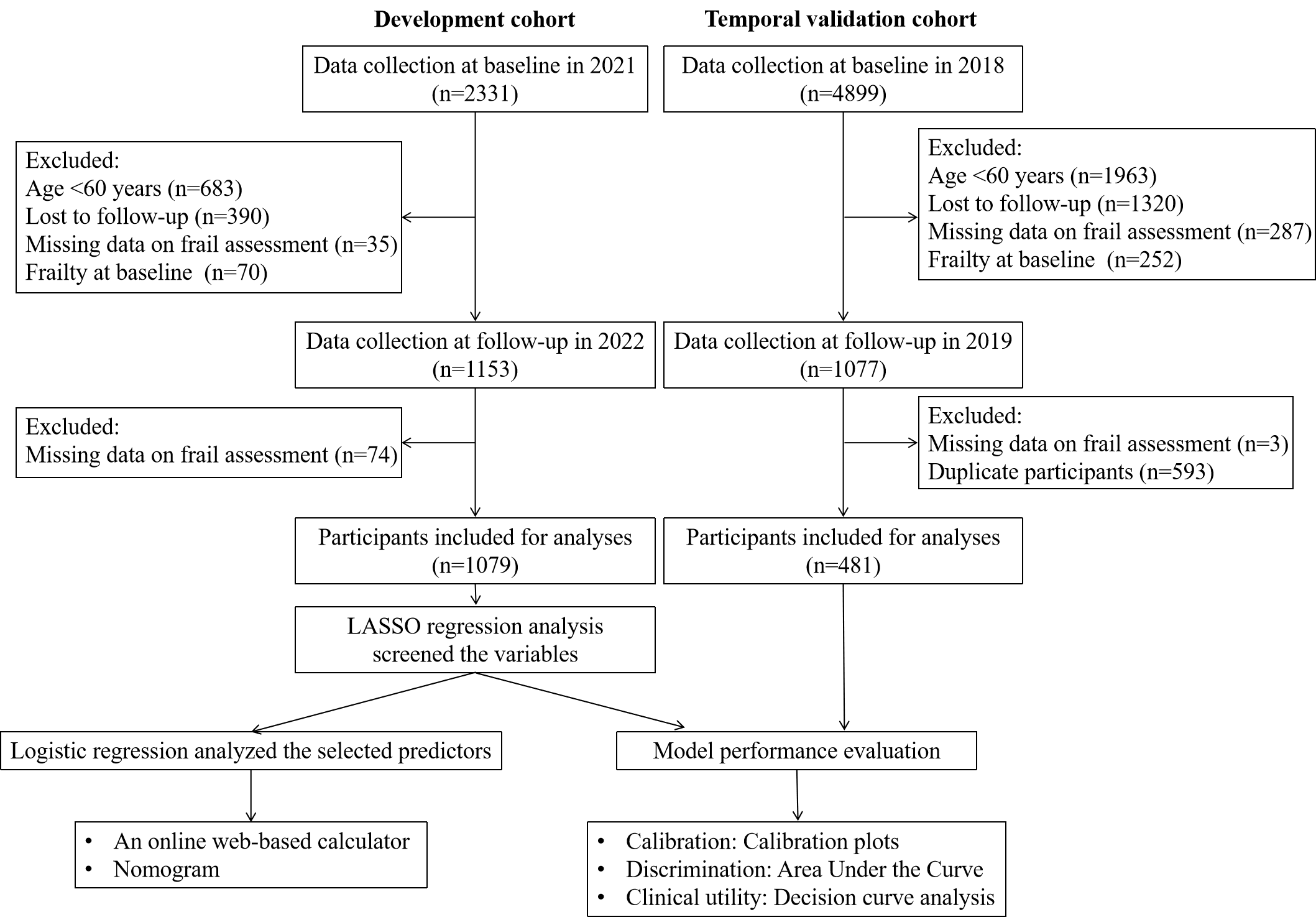


**Supplementary Fig. 1.** Flow chart of development and temporal validation cohort.

**
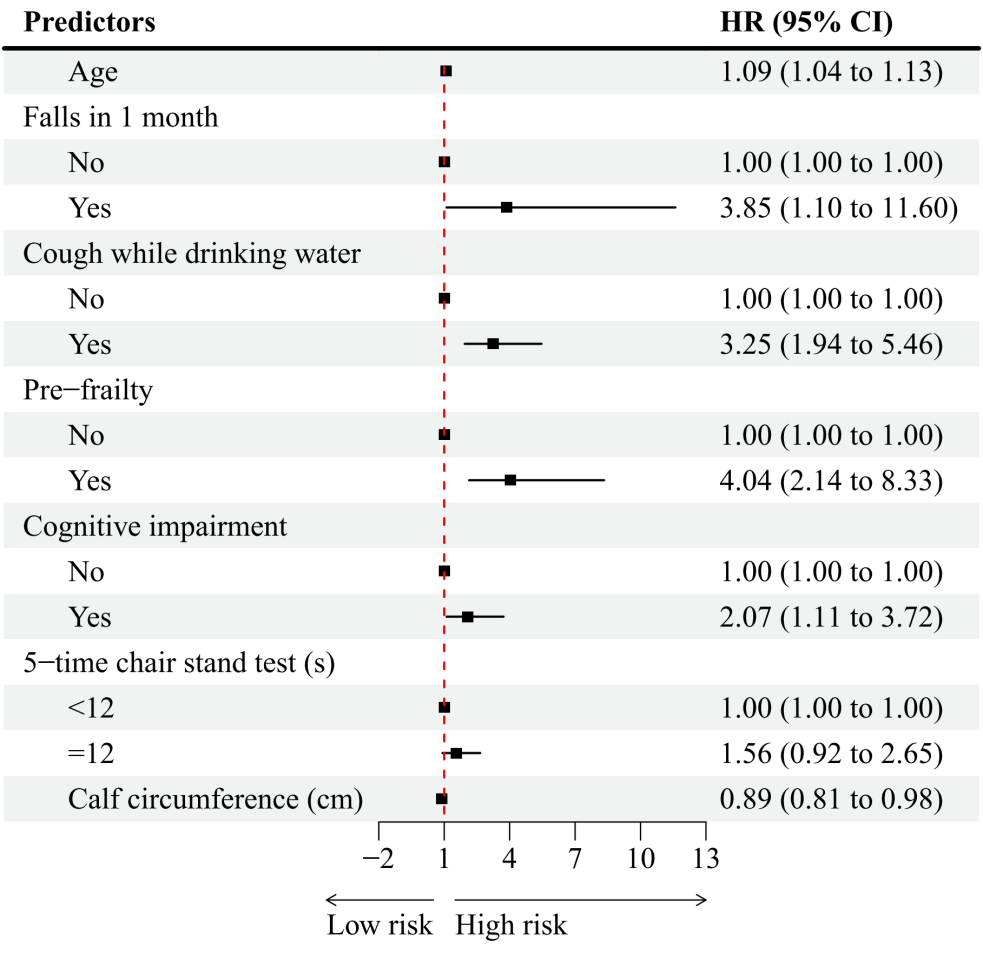
**

**Supplementary Fig. 2.** Logistic analyses of risk factors for incident frailty in the development cohort.

**
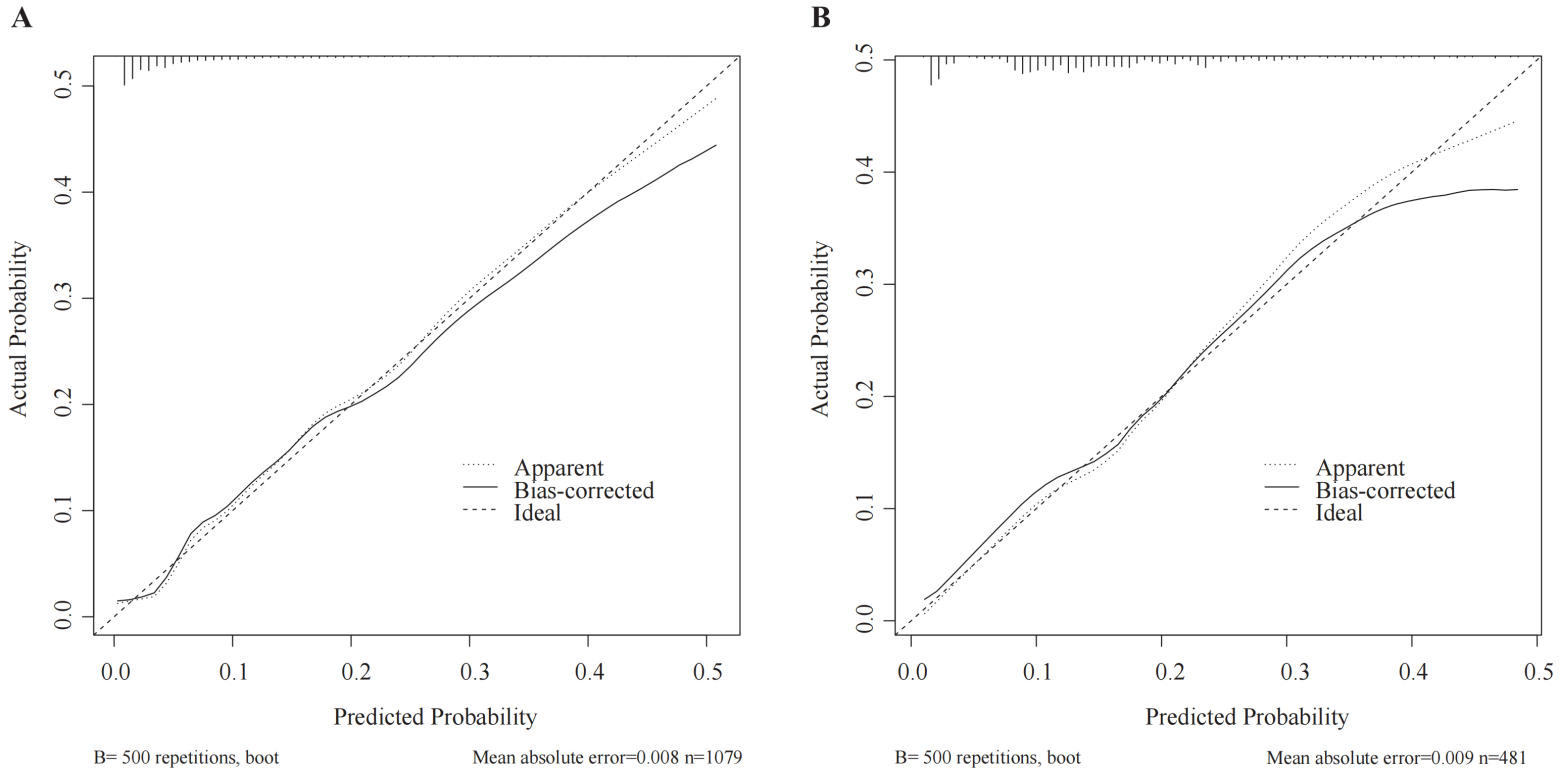
**

**Supplementary Fig. 3.** Calibration plot for the nomogram in the development and temporal validation cohort. (A) calibration plot in the development cohort. The model's calibration indicates excellent calibration power, with a calibration slope of 0.93 and an intercept of -0.14. (B) calibration plot in the temporal validation cohort. The model's calibration indicates excellent calibration power, with a calibration slope of 0.77 and an intercept of -0.34. The dotted line represents the performance of the nomogram, while the solid line corrects for any bias in the nomogram. The dashed line represents the reference line where an ideal nomogram would be situated.


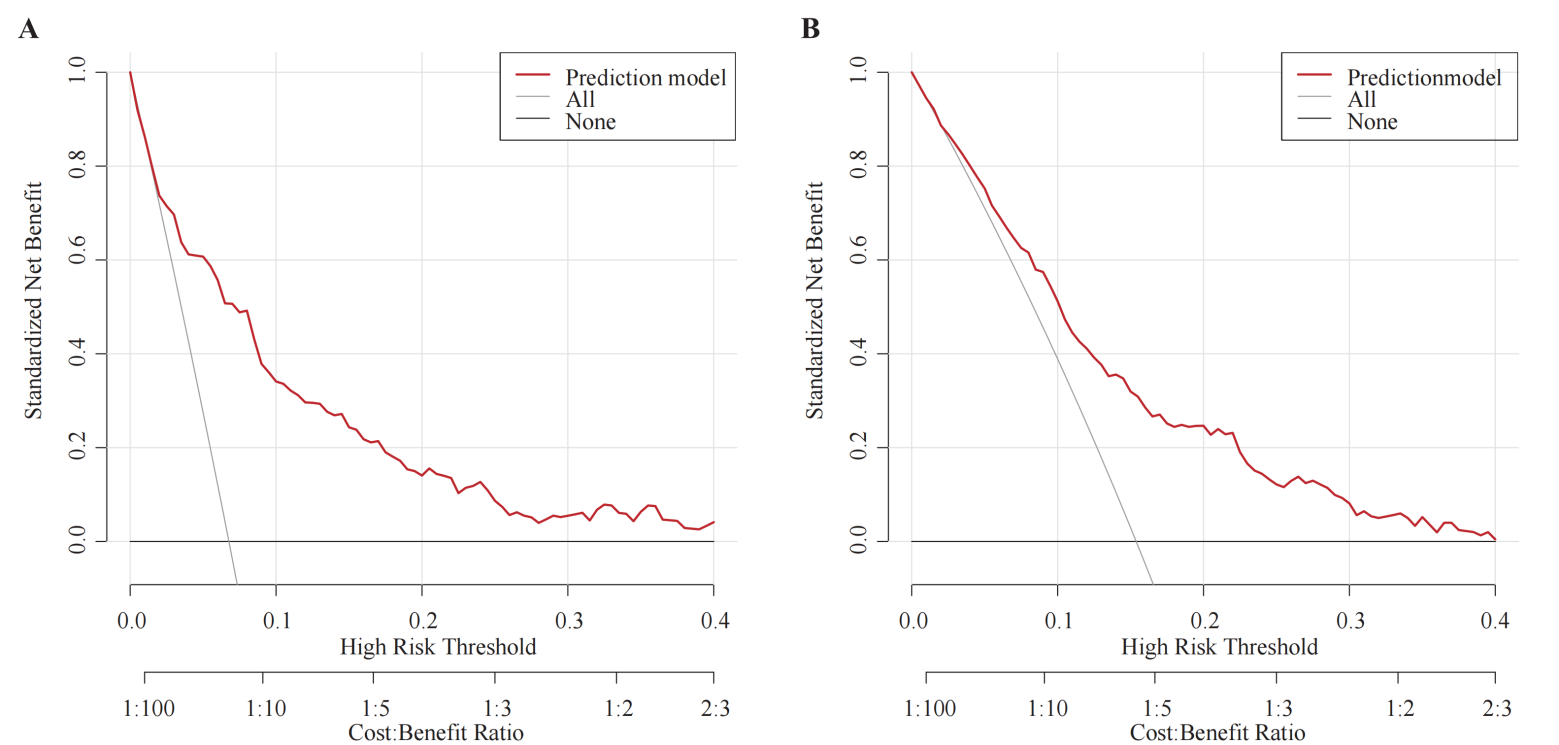


**Supplementary Fig. 4.** Decision curves analysis for the nomogram in the development and temporal validation cohort. Decision curves analysis for the nomogram in the (A) development cohort and (B) temporal validation cohort. The gray line represents the net benefit of the strategy of managing all participants at high risk of frailty development, while the black line illustrates the net benefit of the strategy of treating all participants as if they will not have the probability of developing frailty. The red line indicates the prognostic value of nomogram strategies. As shown, when the threshold probabilities ranged between 4% and 40%, the decision curve analysis suggests that the nomogram provides greater net benefits than managing all participants as if they will or will not develop frailty in the subsequent 12 months in the development and temporal validation cohort.


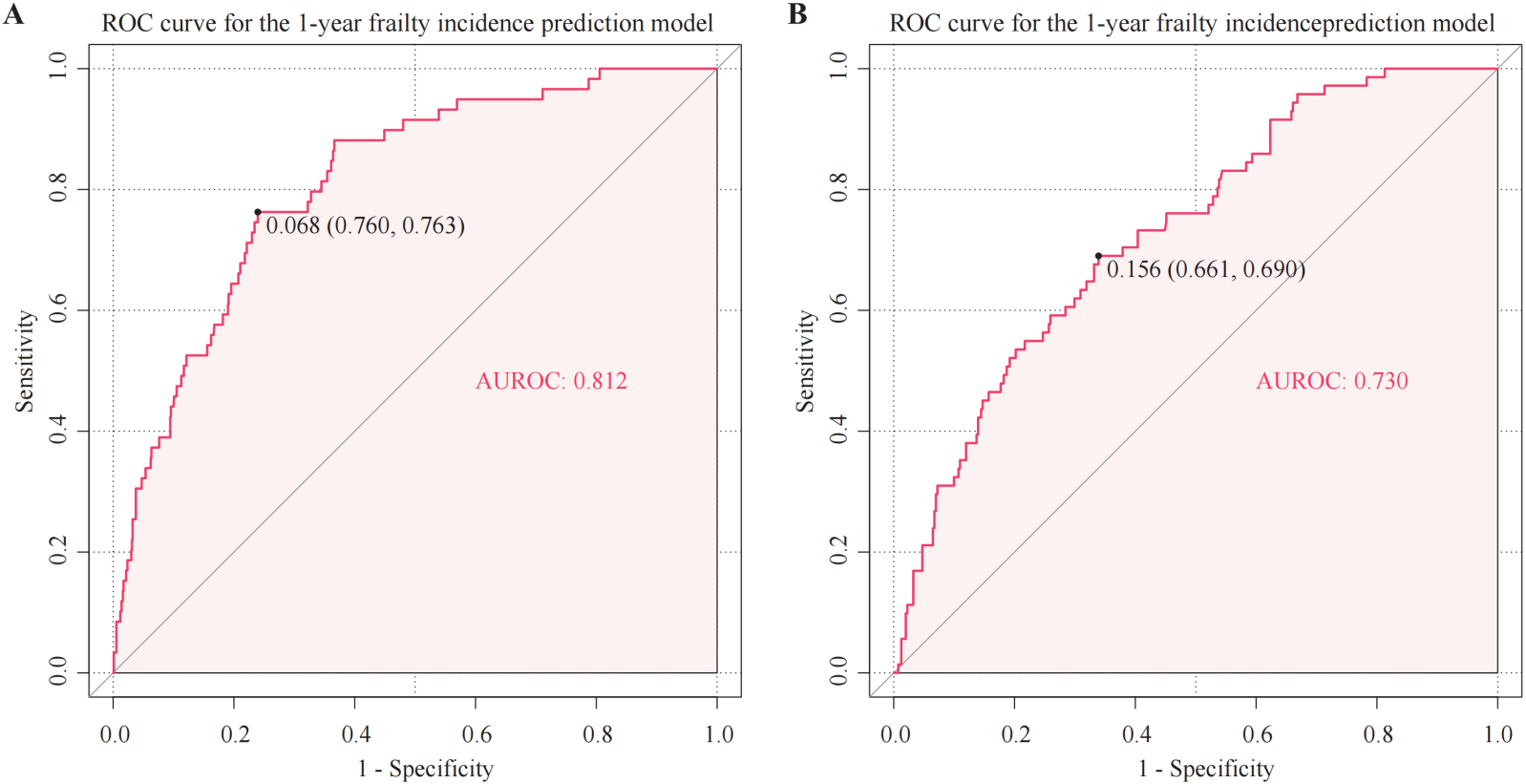


**Supplementary Fig. 5.** The area under the receiver-operator characteristic curve (AUROC) of predicting frailty by excluding participants with follow-up who had missing variables in the development and temporal validation cohort. The ROC curve in the (A) development cohort and (B) temporal validation cohort. The AUROC for predicting frailty was 0.812 in the development cohort (95% confidence interval [CI] 0.759-0.865, p < 0.001) and 0.730 in the temporal validation cohort (95% CI 0.670-0.790, p < 0.001).


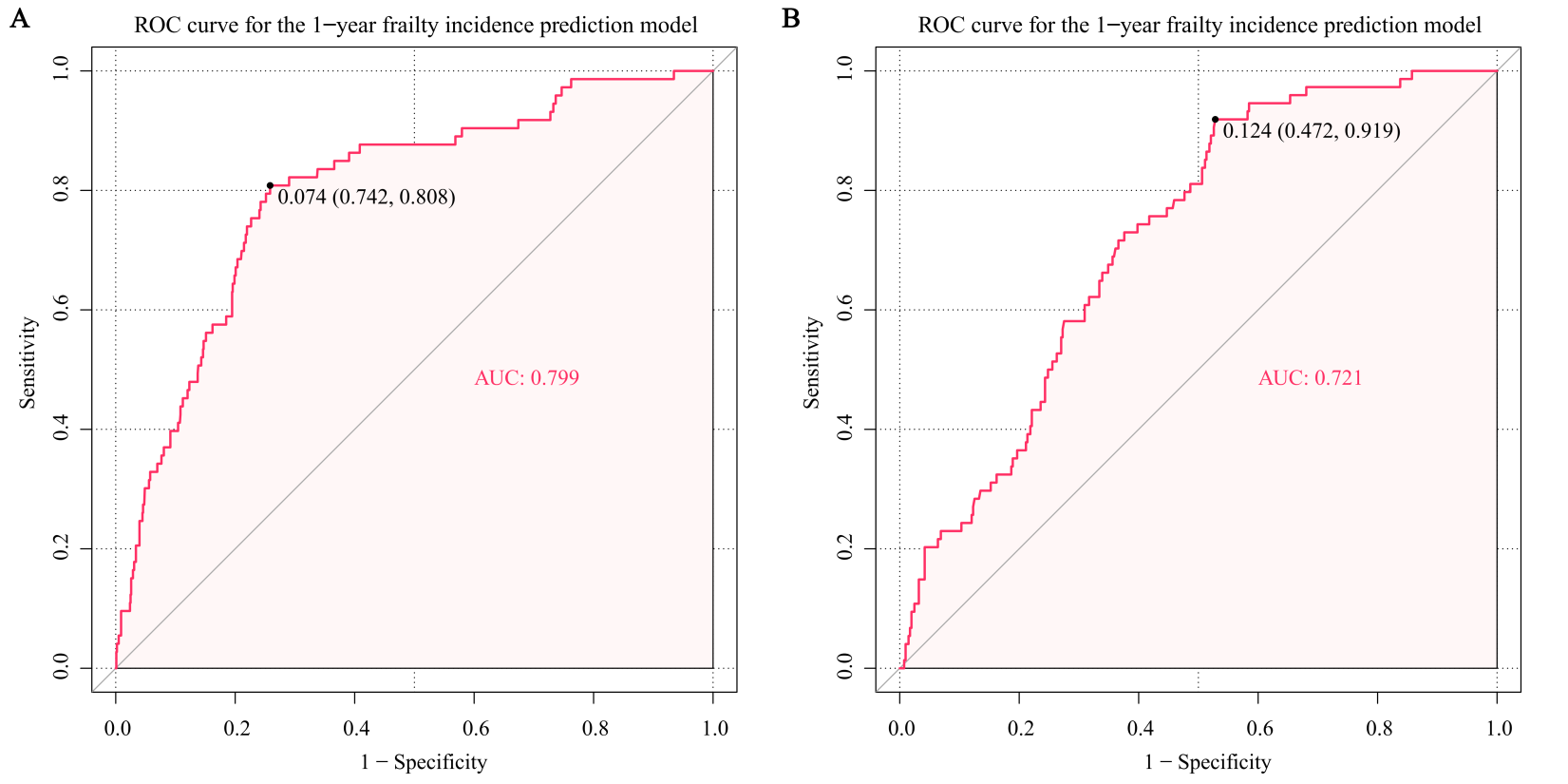


**Supplementary Fig. 6.** The area under the receiver-operator characteristic curve (AUROC) of predicting frailty by excluding participants with follow-up who had missing variables in the development and temporal validation cohort. The ROC curve in the (A) development cohort and (B) temporal validation cohort. The AUROC for predicting frailty was 0.799 in the development cohort (95% confidence interval [CI] 0.746-0.851, p < 0.001) and 0.721 in the temporal validation cohort (95% CI 0.666-0.775, p < 0.001).

**References**

1. Shen Z, Ding S, Shi S, Zhong Z. Association between social support and medication literacy in older adults with hypertension. *Frontiers in public health*. 2022;10:987526. doi:10.3389/fpubh.2022.987526

2. de Waal MW, van der Weele GM, van der Mast RC, Assendelft WJ, Gussekloo J. The influence of the administration method on scores of the 15-item Geriatric Depression Scale in old age. *Psychiatry research*. May 30 2012;197(3):280-4. doi:10.1016/j.psychres.2011.08.019

3. Spitzer RL, Kroenke K, Williams JB, Löwe B. A brief measure for assessing generalized anxiety disorder: the GAD-7. *Archives of internal medicine*. May 22 2006;166(10):1092-7. doi:10.1001/archinte.166.10.1092

4. Pfeiffer E. A short portable mental status questionnaire for the assessment of organic brain deficit in elderly patients. *Journal of the American Geriatrics Society*. Oct 1975;23(10):433-41. doi:10.1111/j.1532-5415.1975.tb00927.x

5. Siotto M, Germanotta M, Guerrini A, et al. Relationship between Nutritional Status, Food Consumption and Sarcopenia in Post-Stroke Rehabilitation: Preliminary Data. *Nutrients*. Nov 15 2022;14(22)doi:10.3390/nu14224825

6. Mollayeva T, Thurairajah P, Burton K, Mollayeva S, Shapiro CM, Colantonio A. The Pittsburgh sleep quality index as a screening tool for sleep dysfunction in clinical and non-clinical samples: A systematic review and meta-analysis. *Sleep medicine reviews*. Feb 2016;25:52-73. doi:10.1016/j.smrv.2015.01.009

7. Fried LP, Tangen CM, Walston J, et al. Frailty in older adults: evidence for a phenotype. *The journals of gerontology Series A, Biological sciences and medical sciences*. Mar 2001;56(3):M146-56. doi:10.1093/gerona/56.3.m146

8. Chen LK, Woo J, Assantachai P, et al. Asian Working Group for Sarcopenia: 2019 Consensus Update on Sarcopenia Diagnosis and Treatment. *Journal of the American Medical Directors Association*. Mar 2020;21(3):300-307.e2. doi:10.1016/j.jamda.2019.12.012
